# Obesity-linked genes may promote prostate cancer among Asian and Hispanic immigrants to North America

**DOI:** 10.3389/fpubh.2026.1742476

**Published:** 2026-02-09

**Authors:** Dan Mercola

**Affiliations:** Department of Pathology and Laboratory Medicine, University of California, Irvine, Irvine, CA, United States

**Keywords:** Asian immigrants, FTO (fat mass and obesity gene), Hispanic immigrants, IGF-1, insulin, North America, obesity-linked gene activation, prostate cancer

## Abstract

**Background:**

Immigration of Asians and Hispanics to countries with higher affluence is associated with a marked increase in the incidence of prostate and other cancers. The goal of this review was to understand the genomic mechanism.

**Methods:**

Cancer incidence, mortality, and comorbidities among Asian and Hispanic immigrants in North America and other affluent countries were systematically reviewed.

**Results:**

Obesity after approximately 10 years or more of acculturation has dramatically increased to levels in some reports that exceed those of males born in North America. The key gene activities associated with obesity include Insulin, FTO (fat mass and obesity gene), IGF-1, and others, leading to a proinflammatory gene expression profile leading to paracrine factors that act on PCa cells and the tumor microenvironment to promote epithelial-mesenchymal transformation, increased invasion, migration, and metastasis.

**Conclusion:**

Obesity among immigrant populations provides a natural experiment that associates obesity with specific obesity-linked genes to suggest the mechanisms of increased prostate cancer.

**Impact:**

Genes associated with obesity are active in periprostatic tissue and promote prostate cancer and progression. Evidence indicates that diet, lifestyle changes, and GLP-1 agonists may be effective therapies with the potential to achieve major medical advances.

## Introduction

It has been reported since the 1970s that the incidence of prostate and other cancers among immigrants from China to the US jumps several fold compared to nonimmigrant Chinese (1–13). Yu et al. reported that the incidence of prostate cancer (PCa) in immigrants from Shanghai to California during the period 1973–1977 increased by over 12 times compared to that in nonimmigrant Shanghai males. The incidence of Colon Cancer has increased 7.1-fold in males and 7.5-fold in female breast cancer. Immigrants from other Asian countries with a low incidence of PCa have exhibited a similar pattern (14). A review of US SEER data found that Asian Indians/Pakistanis living in the United States have a lower incidence of most major cancers compared to US Whites, but a higher incidence than those remaining in the country of origin (14). A similar pattern was found for Chinese immigrants in Alberta Canada (6). For cancers that are traditionally uncommon in China, breast cancer, and PCa, rates for immigrants were also midway between those of non-immigrant Chinese and comparable residents of Alberta (6). Conversely, immigrants to Canada exhibit a healthy Basal Metabolic Index (BMI), which is lost in the first-generation children of immigrants, further emphasizing the effects of acculturation and the environment (13, 15). These observations suggest that Asian immigrants to North America experience a significant increase in prostate and other cancers, possibly due to the influence of environmental factors during acculturation in their adopted country. However, the less efficient detection methods in China and other issues may support this conclusion. Here, additional evidence from mortality data, autopsy data, and similar considerations of Hispanic immigrants to the US is reviewed to support the conclusion of a marked increase in the incidence and severity of PCa among immigrants. Moreover, acquired obesity among immigrants is summarized and shown to be severe, which has not been appreciated in the context of PCa in immigrants, suggesting that specific gene activation associated with obesity may be one basis for the increased PCa and progression in immigrant populations in North America and other affluent countries.

### Incidence of PCa among Asian immigrants

Prior to the widespread adoption of PSA screening, the incidence of PCa in Asian countries may have contributed to a misleading increase in the incidence of prostate and other cancers upon immigration to North America, with more efficient detection (13, 16). Efficient screening was introduced with the PSA test developed by Hybritech of San Diego (acquired by E. I. Lilly & Company) in 1987 but did not gain FDA approval until 1994: the beginning of the “PSA era.” Indeed, soon after the start of the PSA screening era, the PSA screening rate was found to be significantly lower in six Asian countries than in Western countries, which may partially explain the low incidence of PCa in Asian populations during the earlier periods (13). Zhang et al. examined the impact of PSA screening (17). They noted that after the adoption of the American programs for early diagnosis and treatment of PCa, prostate cancers in Asian countries were diagnosed at earlier stages, thereby increasing the incidence in resident populations.

Asian immigrants in the United States and Western Europe, who should have better access to PSA screening, still show a lower incidence of PCa than the native population living in the same regions, indicating that the increased incidence of PCa in immigrants in the United States and Western Europe compared to the non-immigrant Asian population cannot be easily accounted for by the variable use of PSA screening (13, 18).

The incidence of PCa in China in the PSA-era of 2000–2002 was 1.6/100 K, a figure that is ~50-fold smaller than the incidence for Chinese Americans of 80.4/100 K in the same period (Table 1) (17). At the same time, the incidence of PCa among Chinese immigrants of 80.4/100 K was notably lower than that among non-Hispanic Whites, with an incidence of 159.9/100 K. This pattern is similar to the effect of Asian immigration to North Americans in the pre-PSA-era (17).

**Table 1:** Incidence of prostate cancer among immigrants from Asian and Latin American countries to North America*.

Incidence of prosate cancer*	Country of origin	Non-immigrants in target country	Immigrants in target country
			References
China: 1.6, 2.0, 5.3	US: 80.4		
China 2.0, 5.3	Canada, Alberta: 13.1		
5 Asian countries: 42.2–49.8	NSW Australia: 48.8–103.5	159.9	(16, 17)
S. E. Asia (Indonesia, Philippines): 49.8	NSW Australia: 115.4–129.7	44.3	(6, 21)
Iran: 7	Canada, B. C.: 25	119	(21, 22)
Mexico: 29.9	US: 57.2	“	(22)
		96	(19, 20)
		108.9	(38)

*Incidence and mortality figures are per 100,000 people in the PSA era. Ditto marks indicate the use of the value above, which may be from a different reference(S) than that in the row of the ditto mark.

The pattern also holds for immigration to Alberta Canada from China (6). The incidence of PCa in Chinese immigrants increased 6.5-fold compared to the incidence in Shanghai residents (Table 1). As for the US, the incidence did not rise to that of Canadian-born Chinese, which was 3-fold less than that for Canadian-born Chinese in the pre-PSA era (1974–1993) (6).

Iranians who migrated to British Colombia (B. C.) Canada also experienced a rise from an incidence of PCa of 7/100 K to 25/100 K but less than the 96/100 K for the general population of British Columbia (1988–2003) (19, 20).

Australia has a very high incidence (119/100 K) of PCa among Australian-born males (21, 22). Immigrants from five different Asian countries to New South Wales (NSW) Australia exhibit increases in the incidence of PCa from 41 to 87% of the level of the NSW Australian born population (22). Two Asian countries, Indonesia and the Philippines, exhibit larger increases in incidences to 97–109% of that for the locally born population (22). In comparison, the nonimmigrant populations of the five Asian regions exhibited incidences of approximately half the maximum incidence of the respective immigrant groups (Table 1) (21, 22). In summary, the thrust of evidence based on incidence data is that there is a dramatic increase in PCa for the Chinese, other Asian groups, and non-Asian groups upon immigrating to North America as well as to other affluent countries. However, the increase is not at the level of those born in North America or other affluent host European countries. One potential reason for this pattern is that the somatic gene alterations of PCa in Asians and other races vary from those of a European background (23).

### Mortality from PCa among Chinese immigrants

Incidence figures alone do not prove a dramatic rise in PCa and other cancers upon the immigration of Asians and other groups to North America or other affluent countries. Mortality data provided an independent view of the occurrence and progression.

In multiple Asian countries, mortality from PCa increased steadily from 1978 to 1997 (24). PCa mortality in Shanghai increased three-fold from an age-standardized rate (ASR) of 1.61/100 K in 1973 to 4.97/100 K in 2009 (25). The rising trends over time preceded the PSA era, whereas the maximum values were like those within the PSA era. However, Shanghai Chinese immigrants to the US experienced a dramatic increase in mortality to 11–20/100 K (Table 2) (26). In contrast, the mortality due to PCa in the US is considerably higher than the PSA-era Asian values: ~ 45/100 K for US-born European Americans and ~ 75/100 K for US-born African Americans (27–29).

**Table 2:** Mortality of prostate cancer among immigrants from Asian and Latin American countries to North America*.

Mortality due to PCA*	Country of origin	Non-immigrants in the target country	Immigrants in the target country
			References
Shanghai CN: 4.97	US: 11–20		
China: 2.5–4	US: 11		
Japan 5	US: 16.7	45 (African-America 75)	(25–29)
Philippines 11.3	US: 20.4	“	(16, 30)
China: 2.5–4	Canada, Alberta: ~47	“	(16, 26, 27, 30, 148)
Asia: 21	Sweden: 19.4	“	(16, 26, 27, 30)
Mexico: 23	US: 17	“	(16, 148)
		Sweden: 46–103	(31, 34, 35)

*Incidence and mortality figures are per 100,000 people in the PSA era. Ditto marks indicate the use of the value above, which may be from a different reference(S) than that in the row of the ditto mark.

Immigrants from various Asian countries to the US experience greatly increased mortality numbers to 11/100 K for Chinese, 16.7/100 K for Japanese, and 20.4/100 K for Filipinos (Table 2) (30), that is, values 2–5 times that of non-immigrant Asians. However, these increases are less than the mortality rate of the US-born population.

A similar pattern was found among Chinese immigrants in Canada (Table 2) (31). For cancers that are traditionally uncommon in China, such as prostate and breast cancers, mortality rates for immigrants to Canada have significantly increased (6, 32). For PCa, the mortality rate is 47/100 K for Chinese immigrants or a 9-fold increase (6, 32).

Several European countries, such as Sweden, also exhibit a high mortality from PCa of 46–103/100 K (33, 34). Figures are available for immigrants to Sweden from over 55 regions or countries, including China and several Asian countries, indicating that immigrants experience a modest rise in PCa mortality upon immigration to Sweden; however, this is equal to that of Swedes (21).

In contrast, Europeans from the U. K. and Ireland with relatively high mortality from PCa, ASR of 14/100 K, do not change greatly upon immigration to Australia and exhibit a mortality similar to the population born in Australia with an age-standardized rate of 15.1–15.4/100 K (21). This observation may indicate that migration from affluent-to-affluent countries has less impact on immigrants than that from non-affluent to affluent countries such as North America, Sweden, and Australia.

The apparent increase in prostate and other cancers among immigrants is not characteristic of all cancers or diseases. Singh et al. compared cancer mortality for immigrants from seven different but mainly Asian countries to the US and observed substantially higher mortality from stomach, liver, and cervical cancer compared to US-born individuals; however, they had significantly lower mortality from prostate, lung, colorectal, breast, and esophageal cancer, cardiovascular disease, cirrhosis, diabetes, respiratory diseases, HIV/AIDS, and suicide than US-born (30), albeit higher mortality than in the seven Asian countries of origin.

These figures indicate an ~10-fold increase in PCa mortality upon the immigration of Asians to North America (Table 2). This observation supports a possible environmental cause for the increased mortality rate among immigrants that continues today. On the other hand, the increase in mortality for Asian immigrants to a level significantly less than that of the US or Canadian-born population has been argued to be due to the “healthy immigrant effect” (31, 35–37) that is widely observed for immigrants from many countries to countries of high PCa mortality (31, 33, 38). Such a protective effect is incomplete, as the data reviewed here illustrate a significant increase in mortality from PCa upon the immigration of Asians to North America.

Mortality data also have weaknesses, including reliance on death certificates, which may cite a terminal condition rather than the underlying pathology, as well as the rising incidence of PCa across Asia, which may promote an increased disease burden and mortality. Mortality may reflect the increased progression of PCa severity rather than its incidence. Nonclinical PCa, first discovered at autopsy, provides a separate window into its prevalence.

### Incidental/latent PCa and ethnicity

Latent PCa is a cancer found during autopsy in individuals without prior signs or symptoms of PCa. Incidental PCa is often observed in cystoprostatectomy specimens removed for non-prostat-based reasons such as bladder cancer not invading the prostate. The tumors are small and of low grade and are considered to have little malignant potential. The prevalence of latent PCa, discovered by autopsies, is another way to compare the prevalence of PCa among ethnicities and countries. For example, in a review of 23 publications, Rebbeck et al. noted that the overall weighted prevalence of latent PCa was 19.9% in men of Asian, 26.7% in men of European, and 26.2% in men of African descent. This pattern mirrors the incidence and mortality of PCa, which is low in Asian men, high in Scandinavians, and highest in African-Americans (39, 40). The differences were not significant, except when the comparison was for African- and European-descent men combined (26.6%) with Asian-descent men (40).

Regarding incidence and mortality data, the prevalence of latent PCa in several Asian countries has been increasing in recent years. Kimura et al. (16) reviewed 17 studies on the prevalence of latent PCa. Most latent cancer studies reported were on Japanese men, except for a study from Singapore and a study from Iran. Generally, the prevalence of latent cancer in Japanese men for five informative studies indicates an increasing prevalence from 1954 to 2013 (16) in keeping with the prevalence of clinical PCa. However, there was no difference in prevalence between Asians living in Asia and North America (including Hawaii or African Americans vs. Africans) (41).

While there are no studies on the prevalence of latent or incidental PCa among immigrants, the distribution of latent PCa among Asian countries, men of European descent, and African descent agrees with the pattern of increasing incidence of clinical PCa incidence and mortality in these regions.

### Hispanic immigration

A second-largest immigrant population in North America is the Hispanic population, considered here as immigrants from all Latin American countries and the Caribbean. Hispanic immigrants are widely settled in Southwest, California, Washington State, and Canada (42).^1^ As of 2017, Hispanic immigrants accounted for approximately 6.7% of the US population, or ~20 million Hispanic immigrants (43). Although many Hispanics are derived from Mexico, Central America, South America, and the Caribbean, they are broadly an admixture of two major ethnicities: native American ancestry and European, especially Spanish, ancestry (44).

Pinheiro et al. examined the incidence of PCa in large populations of Hispanic immigrants in the PSA-era (1999–2001; Table 2) (38). They observed an incidence of 29.9/100 K for Residents of Mexico but 57.2/100 K for immigrants in the US. The incidence of European Americans (Florida) was 108.9/100 K. Thus, the incidence rate of immigrants is nearly twice that of residents in the country of origin, but about half that of European Americans, which is entirely consistent with the substantial environmental impact that, nevertheless, does not approach the rate of PCa among male US citizens. Pinheiro et al. found 143/100 K for Mexican residents in Mexico, 238/100 K in Mexican immigrants, and 402 in European Americans in Florida. The corresponding mortality rates for all cancers were 116/100 K, 130/100 K, and 182/100 K (38). The mortality from PCa among the three groups, however, did not vary dramatically (range 17 to 23/100 K) (38).

In summary, a review of the experience of Asians and Hispanics upon immigration to the US and other affluent countries shows a very large increase in the recorded incidence of PCa compared to nonimmigrant populations and, with the exception of Hispanics, a sharp rise in the mortality of PCa in the PSA era, which may reflect the increased incidence and/or severity of the disease. These combined results support the environmental effects of the affluence of the host country.

### Obesity in Asian and Hispanic immigrants

Obesity (BMI > 30 where BMI = Weight Kg/height M^2^) is an unfortunate correlation of affluence (45). Obesity is the fifth leading cause of death worldwide.^2^ The World Cancer Research Fund considers that there is strong evidence that being overweight or obese with a BMI > 30 significantly increases the risk of advanced PCa (46). We (47) and others have observed that obesity is linked to PCa, especially the prevalence of aggressive PCa and increased mortality (47–56) although caveats have been expressed [for example, ref. (57)].

Obese Australian men are 2.2 times more likely to develop aggressive PCa than lean men. Each of the 22 pounds of excess weight increased the risk by 40%. Although Asian men tend to be admirably lean, abdominal obesity has been linked to a threefold increase in the risk of PCa in China. In Italy, obesity at the age of 30 has been implicated as a risk factor. In France, mere overweight did not affect the risk, but obesity boosted the odds of PCa by 2.5 times. In Finland, Metabolic Syndrome, which includes abdominal obesity (metabolic syndrome: three or more of a large waistline, high blood pressure, high blood sugar levels, high blood triglycerides, and low HDL cholesterol), appeared to nearly double the risk. Studies of men in California, Cleveland, and Detroit have reported similar results, but several American studies suggest that obesity in youth may be somewhat protective.^3^

It is important to inquire whether the large Asian and Hispanic waves of immigrants to more affluent countries are associated with an increase in obesity. Studies comparing long-standing immigrant populations of 10–15 years in the host country to recent arrivals from the same country are available. For immigrants from many Asian and Latin American countries, increased obesity associated with acculturation in an affluent country has been documented in many studies [reviewed in (42, 58–62)]. Significant increases in obese BMI values among immigrants appear to develop steadily over the 10–15 years post-migration compared to the values of new arrivals and, importantly, for the US, the extent of obesity in some studies even *overtakes* that of the US-born population (or other comparable populations) (3, 63–66). The observations have some generality, as statistically significant increases in obese BMI values have been observed for various immigrant nationalities in different host countries (66–68). In a review of dietary changes after migration to Europe from South Asia (India, Pakistan, Bangladesh, Sri Lanka), the main dietary trend after migration was a substantial increase in energy intake, a reduction in carbohydrates, and a shift from whole grains and pulses to more refined sources of carbohydrates, resulting in a low intake of fiber with an increase in the intake of meat and dairy foods. These high-energy diets may have contributed to the higher observed risks of obesity, type 2 Diabetes and Cardiovascular Disease in immigrants (69, 70). Consistent with this, for seven studies among the immigrant Pakistani community and 24 studies among the indigenous Pakistanis, it was observed that the prevalence of obesity is 10–20 higher among immigrant Pakistanis than among indigenous Pakistanis (71).

In his monograph on living and working with Mexican immigrants in California harvesting various crops for 2 years, Seth Holmes noted that obesity and diabetes were common and major problems among immigrants and sustained with minimal medical management (72). In a large survey of 32,374 individuals, the age- and sex-adjusted prevalence of obesity was 8 among immigrants living in the United States (Asian, Hispanic, foreign-born Blacks) for less than 1 year, but 19% among those living in the United States for at least 15 years (73). The 19% figure is statistically significant for all immigrant subgroups except foreign-born Blacks, indicating that the number of years of residence in the United States that is associated with higher BMI begins after 10 years. The prevalence of obesity among immigrants living in the United States for at least 15 years has approached that of US-born adults (73). Similarly, several studies have observed a significant increase in obesity among Asian and Hispanic immigrants to Canada has been observed in several studies (42, 70). These observations indicate that immigration by Asians, Hispanics, and others to North America and other affluent countries is commonly accompanied by a significant increase in obesity to levels comparable *or greater* than those in nonimmigrant comparable populations of the host country or compared to recently arrived immigrants. Dietary acculturation is expected to explain the occurrence of obesity to levels similar to those of comparable nonimmigrant resident populations; however, it does not readily explain obesity to a greater extent than that of non-immigrant host country populations.

### Obesity as a cause of PCa

In a large meta-analysis of 221 datasets in 141 articles including 282,137 cases, Renehan et al. (74) found an association between increased BMI and the risk of gallbladder cancer, pancreatic cancer, and PCa in men. The International Agency for Research on Cancer Working Group reviewed assessments of the preventive effects of weight control on cancer risk in over 1,000 studies. An increased risk of cancer has been observed in obese subjects (54). Furthermore, it was concluded that there is limited evidence for an association between excess body fat and *fatal* PCa (54).^4^ A large Norwegian cohort of 950,000 men aged 20–74 years was followed for an average of 21 years. While the increase in PCa with increasing BMI was modest, for the older 50–59 years age group who were obese at about age 45 years compared with normal-weight men, a strong association with the incidence of PCa was observed (75). The opposite trend was observed for obese men aged < 60 years (76). In a large study of a European population, the association between obesity and increased PCa-mortality was confirmed and was also observed in the population aged < 60 years (77). These studies of nonimmigrant populations support the role of obesity as a risk factor for PCa.

### Obesity as a cause of aggressive PCa and PCa-associated mortality

Obesity in PCa patients promotes features of aggressive PCa, as indicated by multiple studies in nonimmigrant populations (78–83). Obesity is associated with the progression of PCa to castration-resistant PCa (CRPC), with a poor outcome (84). Obese patients have an increased risk of treatment failure with a short time to biochemical relapse (BCR; elevated PSA following therapy with intent to cure) than non-obese control groups with a BMI ≤ 25 and are at increased risk for developing resistance to chemotherapy (85). Similarly, in patients with PCa treated with radiation, an increased BMI was associated with an increased risk of biochemical failure and distant metastasis (86, 87). In a recent meta-analysis of 23 studies, obesity was associated with a higher risk of death from PCa (79).

However, other large studies have not found an association between BMI and a significant increase in PCa-associated mortality (88) and overall survival (89). A basis for this apparent disagreement has been discussed (88, 90). The effects of hemodilution of serum PSA measurements on the detection of PCa, the low efficacy of DRE in obese patients, treatment bias that disadvantages obesity, frequent and life-shortening comorbidities among the obese such as diabetes and renal failure, and others that may be a frequent cause of death prior to the effects of PCa may reduce the accuracy of PCa-associated mortality figures.

In addition, variables such as body mass index (BMI), which may be analyzed as continuous or categorical variables, may have different results. Freedland et al. (91) carried out a retrospective analysis of 4,268 radical prostatectomy patients within the Shared Equal Access Regional Cancer Hospital (SEARCH) database to examine the association between BMI and PCa-specific mortality (PCSM). In the first analysis, higher BMI was not associated with the risk of PCSM (*p* = 0.112), BCR (*p* = 0.259), or CRPC (*p* = 0.277). However, when BMI was categorized, overweight (hazard ratio (HR) 1.99, *p* = 0.034) and obesity (HR 1.97, *p* = 0.08) were significantly associated with PCSM. Obesity and being overweight were not associated with BCR or CRPC (all *p* = 0.189). On multivariable analysis adjusted for both clinical and pathological features, the results showed little change in that obesity (HR = 2.05, *p* = 0.039) and overweight (HR = 1.88, *p* = 0.061) were associated with a higher risk of PCSM, but not with BCR or CRPC (all p ˃ 0.114). It was concluded that overweight and obesity are associated with an increased risk of PCSM after radical prostatectomy (91).

Overall, compelling evidence suggests that obesity is associated with PCa progression and increased mortality (82, 90).

### Significance

A central observation of this review is that weight gain to the extent of obesity is widespread among Asian and Hispanic immigrants to North America and often exceeds that of the comparable resident non-immigrant population (3, 63–66). This suggests that the known impact of obesity on PCa via a periprostatic inflammatory environment with multiple alterations in gene activity may explain the increased incidence and mortality among these immigrant groups. Many of these gene products promote a range of aggressive PCa phenotypes. These effects of obesity provide a ready-made basis for the increased incidence and aggressiveness of PCa among Asian and Hispanic immigrants.

### Gene expression and other gene alterations associated with obesity

The distribution of body fat in obese subjects may also be important for understanding the origin and progression of PCa in Asian and Hispanic immigrants. The development of visceral and periprostatic fat occurs in proportion to total abdominal fat (92, 93). Fat and obesity-enhanced fat formation due to high-energy diets is regulated by a number of genes, such as fat mass and obesity gene (FTO), Insulin, IGF-1 (Insulin-like Growth 1 Factor) and others. Increased levels of these factors in response to high-energy diets control visceral fat formation. Pathological white fat formation and periprostatic visceral fat formation contain preadipocytes and other cell types (carcinoma-associated fibroblasts, endothelial cells, and M1 macrophages) that secrete adipokines (Leptin, Adiponectin, PAI-1 TNF1, VEGF), chemokines (IL-6, IL-1), and cytokines (CXCL1, CXCL5, CXCL7, and CXCL12). CXCL13, CXCL16, which forms a proinflammatory prostate and PCa microenvironment (51, 94, 95, 96). Many of these gene products act as paracrine factors on tumor cells and stimulate aggressive phenotypic properties (increased proliferation, epithelial-mesenchymal transition (EMT), invasion, migration, and metastasis) (92). Some factors (VEGF and CXCL8) augment angiogenesis, which promotes aggressive growth. Adipocytes, Adipocyte stem cells, stromal fibroblasts, and M1 macrophages enhance inflammation through the elaboration of TNF and CXCL12 that promote inflammation (51, 97).

FTO and certain snp variants of FTO appear to be important in adipocyte hyperplasia and lipogenesis in obesity (98, 99). FTO expression and activity are associated with obesity, body weight, fat mass, and BMI (100). In pigs, FTO protein levels in the Islets of Langerhans are associated with energy intake (98, 101). Expression was low on a low-energy diet and high on a high-energy diabetogenic diet. FTO levels in the pancreas may directly influence insulin release from beta cells during a high-energy diet (101). Insulin is a major driver of adipocyte proliferation and hyperplasia. FTO may also act directly on fat tissue. Fat pads from FTO-expressing mice fed a high-fat diet showed more adipocytes than controls (102). FTO influences adipogenesis by regulating events during mitotic clonal expansion. The effect of FTO on adipogenesis appears to be mediated by enhanced expression of the pro-adipogenic short isoform of RUNX1T1 (RUNX1 Partner Transcriptional Co-Repressor 1), which enhances adipocyte proliferation and is increased in FTO-expressing mouse embryonic fibroblasts (MEFs) and reduced in FTO-KO MEFs. Similarly, FTO overexpression in the liver promotes lipogenesis and lipid droplet enlargement and suppresses CPT-1e (carnitine palmitoyltransferase 1e)-mediated fatty acid oxidation via the SREBP1c (Sterol Regulatory Element Binding Protein 1c) pathway, promoting excessive lipid storage and nonalcoholic fatty liver disease (NAFLD) (99). FTO also enhances preadipocyte differentiation through the CCAAT enhancer binding protein *β* (C/EBPβ) pathway and facilitates adipogenesis and fat deposition by altering the alternative splicing of RUNX1T1, expression of peroxisome proliferator-activated receptor *γ* and ANGPTL4 (Angiopoietin-like 4), and phosphorylation of PLIN1 (Perilipin 1). FTO inhibits lipolysis by inhibiting IRX3 (Iroquois homeobox 3) expression and the leptin pathway. The cumulative effect is adi[pogensis and adipocyte hyperplasia–the development of obesity].

Insulin complements these effects by stimulating adipogenesis and lipogenesis through the induction of SREBP-1c (SREBP-1, sterol regulatory element binding protein 1) and several other transcription factors involved in human adipocyte differentiation (103–105). Another important action of insulin in fat cells is to limit lipolysis by inhibiting HSL (Hormone-Sensitive Lipase) thereby maintaining fat mass.

Increased visceral fat in the environment of the tumor-bearing prostate (periprostatic visceral fat) may contribute to aggressive cancer (51, 106). In a detailed review (51), Saha et al. identified five principal cell types of periprostatic white adipose tissue that secrete over 13 key factors that act on 22 signaling pathways to promote remodeling of the extracellular matrix, support neovascularization, recruit immunosuppressive cells, and induce EMT, and thus tumor cells with increased migratory properties and metastatic potential. Adipocytes of the periprostatic adipose tissue play a major role in secreting seven key obesity-linked factors that increase the phenotypes of aggressive PCa (51). The obesity-linked genes include Il-6, TNFα, IL-1β (107) Adiponectin, and Leptin (106, 108–110). Paracrine stimulation leads to the activation of the PI3K-AKT–mTOR pathway in tumor cells, promoting proliferation, migration, and invasion, as well as other pathways of survival (e.g., AMPK, STAT3), EMT (STAT3), inflammation (NF-κB), and metastases (STAT3, CXCL1).

Chen et al. (111) carried out a bioinformatics analysis using multiple publicly available databases and found that four genes, MSMB (Microsemino protein beta), BMP5 (Bone morphogenetic protein 5), THBS4 (Thrombospondin 4), and POPDC3 (Popeye Domain Containing 3), may lead to prostate carcinogenesis in patients with obesity. MSMB and THBS4 affect PCa progression, and all four genes are risk factors for CRPC development. These obesity-related genes were also correlated with immune cells and immune cell infiltration in PCa. MSMB was downregulated in PCa CRPC and MSMB decreased PCa cell proliferation. MSMB may be essential for PCa development in obese individuals. These genes are candidate genes for the development and progression of PCa in Asian and Hispanic Immigrants.

FTO may also regulates the expression of HOX13B (Homeobox B13) in the adult (112, 113, 114). During development, HOXB13 participates in the androgen receptor (AR) to regulate AR target genes during the development of the prostate and its secretory functions (115). In adult, elevated HOXB13 activates proliferation in endometrial and gastric cancers via activation of the Wnt pathway and augments tumor invasion and metastases (112, 114). These observations suggest that FTO may favor gene expression changes in the prostate that promote cancer progression in endometrial and gastric cancers.

Certain somatic gene alterations in PCa may be linked to obesity in PCa (116, 117). The TMPRSS2: ERG translocation fusion rearrangement places an androgen receptor-sensitive sequence, TMPRSS2, upstream of the ERG coding gene sequence, whose gene product is a transcription factor affecting many other genes (118, 119). The translocation is found in ~50% and up to 75% of PCa cases (120) although this has been challenged (121). TMPRSS2-ERG fusion can be an early change that can be detected in a proportion of high-grade intraprostatic neoplasia (HGPIN) lesions and may precede chromosome-level alterations, including copy number changes, which are considered on the path to invasive PCa and may be a PCa-causing mechanism (122, 123, 124).

Based on a large prospective study of PCa patients (*n* = 1,243, 92% white) from the Physicians’ Health Study Professionals Follow-Up study, Peterson et al. found positive associations between both generalized obesity and central obesity and the risk of PCa metastases and death in patients with TMPRSS2-ERG-positive tumors but not among obese men with TMPRSS2-ERG-negative tumors (116). Each 5-unit increase in BMI before diagnosis in men with TMPRSS2-ERG-positive tumors was associated with a proportionally higher risk of lethal disease (HR 1.48, 95% CI 0.98–2.23). Each 8-inch increase in waist circumference before diagnosis was associated with an increased risk of lethal disease (HR 2.51, 95% CI 1.26–4.99). These two observations were not observed in patients with TMPRSS2-ERG-negative tumors. However, obesity was not related to PCa outcome of PCa (116).

Egbers et al. studied 2,208 patients and found an inverse relationship between obesity and PCa (117). In this study, over half of the patients had a low Gleason Score of <6, and predominately had low stage T2 disease. This finding is consistent with the finding that obesity is associated with *advanced* PCa and death (49).

Ribeiro et al. I identified significant differentially expressed genes for lean compared to overweight, obese, and those with extra prostatic cancer versus organ-confined PCa or benign prostatic hyperplasia (87). Thirty-four differentially expressed genes were identified. Several genes suggested an antilipolytic and adipo/lipogenic gene expression profile, thereby maintaining or augmenting the obese state. The anti-apoptotic genes ANGPT1 (Angiopoitin-1) and HSPB8 (Heat Shock Protein Family B Member 8) were upregulated in the periprostatic tissue of overweight/obese subjects. At the same time, the expression of LEP and ANGPT1, which promote endothelial, mesenchymal, and tumor cell growth and differentiation, was increased. This pattern suggests angiogenesis, increased cell growth, and an anti-apoptotic gene product impacting cells in the periprostatic adipose tissue of overweight and obese men. Moreover, overexpressed genes are involved in innate and adaptive components of the immune system, including LEP, which upregulates both innate and adaptive immunoinflammatory responses; NPY1R, whose product inhibits cell activation; and CYSTLR2 (Cysteinyl Receptor 2), whose product increases pro-inflammatory cytokine expression. FADS1, whose gene product stimulates the expression of inflammatory mediators [prostaglandin E2, PGE2, thromboxane A2, TXA2, and leukotriene B4 (LTB4)], Eukaryotic Translation Initiation Factor 5A, EIF5A, essential for Nitrous Oide Synthase-2, NOS2, and translation, are both downregulated in the periprostatic adipose tissue of obese or overweight men. This pattern observed in the periprostatic tissue of obese or overweight men suggests that the microenvironment is modified toward hypercellularity and immunosuppression, thereby favoring PCa progression.

High expression of three of the four studied snps of the fatty acid synthase (FASN) gene is associated with several aggressive cancers, including aggressive PCa (125, 126). Primary prostate tumors with ERG rearrangement show increased FASN expression. Expression was also significantly increased in prostate tumors from carriers of the HOX13B G84E mutation compared to matched controls. The expression level correlated with a BMI (126, 127). Thus, the effects of FASN expression appear to cooperate with the known features of obesity that contribute to aggressive disease. Small molecule FASN inhibitors are in development, with new candidates in clinical trials (ClinicalTrials.gov, NCT02595372).

### Function

Numerous cells and gene products in periprostatic adipose tissue are linked to aggressive phenotypes. A growing body of literature on mouse models has demonstrated their functional roles in PCa progression. This includes migration of periprostatic adipose stromal cells [ASCs, also known as adipose tissue resident mesenchymal stromal cells (MSCs)] into adjacent tumors under the influence of CXCL1, expression of the MSC marker *α*-smooth muscle actin (αSMA), and participation in tumor growth and vascularization, which do not occur in lean mice (128–130). Other stroma-recruited tumor cells also promote metastases. Here, CXCL16, a ligand for CXCR6, facilitates mesenchymal stem cell or very small embryonic-like cell recruitment into prostate tumors. CXCR6 signaling stimulates the conversion of mesenchymal stem cells into CAFs which secrete SDF-1 (stromal-derived factor-1, aka CXCL12). CXCL12 binds to CXCR4 in tumor cells and induces EMT, which leads to tumor metastasis (131). Saha et al. (51) provide a reference table of 16 periprostatic adipose tissue-secreted proteins and their experimental effects on one or more aggressive phenotypes of PCa that include most of the gene products discussed above.

The functional effects of periprostatic adipose tissue that promote PCa aggression in mouse models have been summarized (51, 132).

Houthuijzen et al. (133) reviewed studies provided functional evidence of mesenchymal stem cell homing to tumors, where they modulate the immune system and facilitate tumor growth, angiogenesis, and metastasis. Recent studies have shown that MSCs also play a significant role in resistance to various anticancer drugs. The functional properties of MSCs in tumor progression and drug resistance have been summarized (133). These results have not been confirmed in obese versus lean Asian or Hispanic immigrant PCa patients.

### Summary

Taken as a whole, for very large populations of different immigrant ethnicities, the increased obesity of these groups may be correlated with increased expression and/or alterations of genes of periprostatic fat and with experimental evidence for roles in the promotion of PCa and roles in aggressive PCa in immigrant populations, suggesting an immigration-obesity-gene activation-prostate cancer axis as the mechanism of increased incidence and aggression of PCa in Asian and Hispanic immigrants to affluent countries. This is an extension of the role of obesity as a causal agent for PCa and aggressive PCa in other obese populations. This extension depends on the observation that obesity is indeed a notable feature of both Asian and Hispanic Immigrants, as reviewed here. Functional studies have supported the role of obesity-linked genes in promoting PCa development. However, there is little evidence that the genes of the proinflammatory microenvironment of prostate function promote PCa and aggressive PCa in immigrants. However, an ongoing Prospective Multiethnic Cohort Study exploring obesity, genes, lifestyle, and cancer risk among various ethnic and immigrant groups may provide insight (134).

### Caveats remain

FTO expression is low in most PCa cases surveyed in TCGA database (135). FTO stabilizes DDITA4 expression, which participates in cell proliferation, migration, and bone metastases, according to TCGA data. RNA-seq revealed that DDIT4 expression was markedly upregulated in the bone metastasis of this group of cases and that DDIT4 participates in the PI3K/AKT–mTOR signaling pathway, which promotes proliferation. FTO and DDIT4 levels are correlated with other markers of bone metastases such as NOTCH1/BAP1/TNFSF11 (135). The extent of obesity was not assessed in this study.

Moreover, not all FTO-stabilized gene products may activate the PI3K/AKT–mTOR pathway or promote progression. FTO-stabilized miR-139–5p (an expressed noncoding micro RNA 139-5p that binds genes, some of which are associated with breast cancer) targets ZNF217 (zinc finger family protein 217) to suppress PCa cell malignancies by opposing the activation of the PI3K/Akt/mTOR signaling pathway (136).

Other FTO-stabilized gene products are associated with the suppression of PCa. The stabilizing effects of FTO are due to its demethylase activity which demethylates 6-methyl-adensoince groups from 3’-UTR sites of mRNA thereby depriving “reader” proteins from binding and participating in the transport and destruction of the demethylated form of the RNRNA. Thus, FTO suppressed PCa progression by maintaining CLIC4 (Chloride Intracellular Channel 4) mRNA stability (137).

Other studies do not support obesity as a cause of PCa or promote aggressive PCa (76, 88, 89).

### Treatment

Numerous possible molecular therapeutic targets have been identified. Management by individual or combined targeting may not be practical for a large number of potential therapeutic targets. Recent studies have indicated that dietary- and lifestyle-based regimens may be promising. The only phase III randomized clinical trial on a therapeutic diet for PCa is an intervention that encouraged increased use of vegetables reported in January 2020, which showed no impact on the progression of PCa (138) consistent with the analysis of 15 prospective studies on the consumption of fruits and vegetables. Had no significant effect on the risk of PCa (139).

However, a small intervention study on PCa patients based on lifestyle changes involving the combination of meditation, exercise, and a low-calorie diet reported weight loss, waste reduction, other positive phenotypic changes, and corroborating gene expression changes (140–142).

Freedland et al. conducted a small 43 subject randomized multi-center trial of PCa patients who were initiating androgen deprivation therapy (ADT), a therapy that may promote insulin resistance. A low-carbohydrate diet plus walking at least 30 min, 5 days a week, was compared to standard diet and exercise habits in control patients. The low-carbohydrate walking regimen reduced insulin resistance over the study period, suggesting that future larger studies of the regimen for reducing ADT-induced disturbances may be effective (53).

In this observational study, a pre-post design was employed, in which each patient served as his own control. PSA rise rates were determined for each patient during two periods: from post-treatment recurrence to study start (“prestudy”), and from immediately before the intervention (baseline) to its end (0–6 months). There was a significant decrease in the rate of PSA increase from pre-study to 0 to 6 months (*p* < 0.01). Four of the 10 evaluable patients experienced an absolute reduction in PSA levels over the entire 6-month study. Nine of the 10 patients had reduced rates of PSA rise and improved PSA doubling times. Median PSA doubling time increased from 11.9 months (prestudy) to 112.3 months (intervention). These results provide preliminary evidence that the adoption of a plant-based diet in combination with stress reduction may attenuate disease progression and have therapeutic potential for the clinical management of recurrent PCa (143). The immigrant populations were not explicitly considered.

Recent evidence from several large observational studies indicates that plant-based diets have a significantly favorable impact on PCa progression (144–146). In a prospective study of 3,505 PCa patients, the median time from diagnosis/treatment to the quality-of-life questionnaire was 7.0 years. A higher plant-based diet index was associated with better sexual function, urinary irritation/obstruction, urinary incontinence, and hormonal/vitality scores. Consuming healthy plant-based foods was also associated with better sexual and bowel function, as well as urinary incontinence and hormonal/vitality scores in the age-adjusted analysis, but not in the multivariable analysis. The results provided supportive evidence that greater consumption of healthy plant-based foods is associated with modestly higher scores in quality-of-life domains among patients with PCa. However, the effect on BMI remains unstudied.

Recently, encouraging results of plant-based diet consumption on the progression of PCa in the CaPSURE program were described (144). From 1999 to 2018, 2022 participants from 43 urology practices across the US, representing multiple ethnic backgrounds, were included. A higher intake of plant foods after PCa diagnosis is associated with a lower risk of cancer progression. Increasing plant-based diet components also reduces the risk of other urologic cancers (bladder and kidney) and aligns with environmental goals (147). The regimen’s impact on BMI remains unstudied. This study did not include targeted recruitment of immigrants.

Interestingly, if a plant-based diet and other energy restrictions are applied to immigrant populations, the regimen would restore dietary practices toward those of the countries of origin.

## Conclusion

Male Asian and Hispanic immigrants to North American and likely most affluent countries undergo acculturation often leading to obesity with a prevalence equal to or exceeding that of the resident male population, itself a serious public health problem. In obesity many obesity-linked genes of cells of the periprostatic environment are altered in activity leading to products that may initiate prostate cancer and/or stimulate an aggressive phenotype: the immigration-obesity-gene activation-PCa axis. Recent studies indicate that lifestyle interventions, including a combination of a plant-based diet, exercise, meditation as well as medication, have the potential to reduce BMI values, which may be associated with reduced periprostatic inflammation and reduced adverse gene expression, thereby reversing gene expression changes that promote PCa and aggressive PCa. Demonstration of these benefits in Asian and Hispanic immigrants would support the hypothesis that the immigration-obesity-gene activation-PCa axis is central and would provide a major advance in cancer care.

## Footnotes

1
http://cervantesobservatorio.fas.harvard.edu/sites/default/files/hispanic_map_2017en.pdf

2
https://www.who.int/health-topics/obesity#tab=tab_1

3
https://www.health.harvard.edu/newsletter_article/obesity-and-prostate-cancer#:~:text=Studies%20from%20around%20the%20world,weight%20boosted%20risk%20by%2040%25

4
http://www.wcrf.org/sites/default/files/Prostate-Cancer-2014-Report.pdf
